# Central integration and neural control of blood pressure during the cold pressor test: a comparison between hydrochlorothiazide and aliskiren

**DOI:** 10.14814/phy2.12502

**Published:** 2015-09-14

**Authors:** Sara S Jarvis, Yoshiyuki Okada, Benjamin D Levine, Qi Fu

**Affiliations:** 1 Institute for Exercise and Environmental Medicine, Texas Health Presbyterian Hospital DallasDallas, Texas; 2 University of Texas Southwestern Medical CenterDallas, Texas; 3 Department of Biological Sciences, Northern Arizona UniversityFlagstaff, Arizona

**Keywords:** Muscle sympathetic nerve activity, renin inhibition, thiazide diuretic

## Abstract

Individuals with hypertension and sympathetic overactivity are at risk for cardiovascular events. Renin inhibitors are new while thiazide diuretics are first-class drugs used for treatment of hypertension. The purpose of this study was to determine whether 6 months of treatment with aliskiren (ALSK) or hydrochlorothiazide (HCTZ) would alter blood pressure (BP) and muscle sympathetic nerve activity (MSNA) indices in older mild hypertensives during a cold pressor test (CPT). We hypothesized that the ALSK group would demonstrate a blunted response compared to HCTZ. Nineteen (9 men, 10 women) subjects performed a CPT pre- and post treatment where heart rate (HR), systolic BP (SBP) and diastolic BP (DBP), and MSNA were measured. Blood samples were withdrawn for assessment of renal-adrenal hormones. Both medications lowered ambulatory SBP and DBP (*P *<* *0.05). Direct renin tended to be higher in the ALSK group after treatment (*P *=* *0.081). Aldosterone was higher in the HCTZ group after treatment (*P *<* *0.001). As expected, both groups showed increases in HR, SBP, DBP, and MSNA during the CPT (all *P *<* *0.05). All cardiovascular and MSNA responses were similar pre- and post treatment in both groups (peak CPT SBP: 26 ± 10 vs. 17 ± 21 and 21 ± 20 vs. 29 ± 15 mmHg for pre vs. post for HCTZ and ALSK, respectively; peak CPT MSNA burst frequency: 13 ± 8 vs. 11 ± 11 and 11 ± 17 vs. 6 ± 13 bursts/min; all *P *>* *0.05). Treatment with these antihypertensive medications lowered BP but was not successful in lowering the responsiveness to the CPT.

## Introduction

Individuals with hypertension and sympathetic overactivity are at increased risk for the occurrence of cardiovascular events (Grassi 2009). Older hypertensive patients possess both of these characteristics, making them vulnerable to cardiovascular events. Treatments that effectively lower blood pressure (BP) are beneficial for both morbidity and mortality outcomes in these patients. However, some studies have indicated that, while BP was lower after antihypertensive medication treatment, some medications still allowed for persistent sympathetic overactivation (Lindqvist et al. 1994; Fu et al. 2005; Menon et al. 2009). This raises an interesting concern as to the long-term beneficial effects of some antihypertensive medications. Thus, with the many different types of antihypertensive medications available, all targeting different and specific pathways, it is important to understand how BP is lowered in hypertensive patients and also whether these medications favorably alter physiological responses, particularly to hypertensive stimuli.

Thiazide diuretics (e.g., hydrochlorothiazide, HCTZ) are first-class antihypertensive drugs that are commonly prescribed in the clinic. Thiazide diuretics lower BP by reducing blood volume through the inhibition of sodium and chloride ion reabsorption (Shah et al. 1978). Renin inhibitors (e.g., aliskiren, ALSK) are relatively new in the treatment of hypertension. These drugs lower plasma renin activity, which subsequently lowers both angiotensin I and II. A reduction in angiotensin I and II thereby decreases the vasoconstrictive effect of angiotensin II and lowers BP (Nussberger et al. 2002). Some studies have shown that thiazide diuretics may activate the renin-angiotensin-aldosterone system (RAAS) and increase muscle sympathetic nerve activity (MSNA) (Lijnen et al. 1981). Conversely, direct inhibition of renin has been shown to lower MSNA (Siddiqi et al. 2011).

We previously examined responses to 20 min of upright tilt in older patients taking either HCTZ or ALSK to determine whether these medications altered the responses to upright tilt (Okada et al. 2013). We reported that treatment with HCTZ increased MSNA in both resting conditions and during upright tilt, when compared to renin inhibition (ALSK), which reduced upright MSNA (Okada et al. 2013). Additionally, there was an upregulation of the RAAS with HCTZ treatment where the increase in upright MSNA induced by the drug treatment was positively related to the increase in aldosterone (Okada et al. 2013). These results indicate that treatment with a thiazide diuretic leads to sympathetic activation through an upregulated RAAS, despite BP being well controlled.

While examining the efficacy of BP-lowering medications, an important consideration would also be to examine responses to sympathetic activation such as to the cold pressor test (CPT) – a powerful sympathetic activator. That is, does antihypertensive treatment lessen the magnitude of this response? In normotensive individuals, a magnified response (i.e., hyperreactivity) could be indicative of the future development of hypertension (Krantz and Manuck 1984; Treiber et al. 2003). For example, it has been reported that augmented responses to the CPT may predict the development of hypertension (Menkes et al. 1989; Kasagi et al. 1995). In hypertensive individuals, this hyperreactive response may prove to be problematic when we begin to consider activities that further increase BP or environments (e.g., cold) that might induce a sympathetic response. The current study sought to determine whether treatment with HCTZ or ALSK would alter central integration and the efferent pathway of neural control of BP during a CPT. As we previously demonstrated that treatment with a thiazide diuretic may enhance MSNA and treatment with a renin inhibitor may depress MSNA (Okada et al. 2013), we hypothesized that patients taking ALSK would demonstrate a blunted response due to a suppression in sympathetic outflow when compared to those taking HCTZ where sympathetic outflow might be enhanced.

## Methods

### Subjects

Twenty-two (11 men, 11 women) elderly patients with mild hypertension (systolic BP 140–159 mmHg and/or diastolic BP 90–99 mmHg) volunteered for this study. Descriptive characteristics of the subjects are outlined in Table 1. Exclusionary criteria were: cardiopulmonary, neurological and renal disease, diabetes mellitus, secondary hypertension, significant medical history, smoking, recreational drug use, hormonal contraceptive use within the previous 6 months, BP above 160/100 mmHg, body mass index >30 kg m^−2^, and history of gouty arthritis. All subjects gave written informed consent to participate in the study which was approved by the Institutional Review Boards at the University of Texas Southwestern Medical Center and Texas Health Presbyterian Hospital Dallas. This study followed guidelines set forth in the *Declaration of Helsinki*.

**Table 1:** Subject characteristics

	HCTZ (*n* = 10, 5M/5F)		ALSK (*n* = 9, 4M/5F)	
	PRE	POST	PRE	POST
Age (years)	68 ± 6	68 ± 7	66 ± 4	67 ± 4
Height (cm)	169.7 ± 8.7	169.7 ± 9.2	167.6 ± 10.9	167.9 ± 11.0
Weight (kg)	75.5 ± 9.8	74.7 ± 10.6	76.9 ± 14.3	77.0 ± 15.5
Body mass index (kg m^−2^)	26.1 ± 1.9	25.8 ± 1.6	27.2 ± 2.7	27.1 ± 3.1
ABPM SBP (mmHg)				
Awake	149 ± 7	131 ± 8*	143 ± 9	131 ± 7*
Sleep	129 ± 6	113 ± 10*	127 ± 14	113 ± 13*
ABPM DBP (mmHg)				
Awake	83 ± 8	76 ± 7*	78 ± 5	74 ± 6*
Sleep	70 ± 6	63 ± 6*	69 ± 10	62 ± 7
Hematocrit (%)	40 ± 4	39 ± 4*	41 ± 4	39 ± 4*
Total hemoglobin mass (g)	570 ± 141	580 ± 142	609 ± 195	609 ± 160
Total blood volume (mL)	4731 ± 810	4863 ± 861	4822 ± 1233	4984 ± 999
Red cell volume (mL)	1723 ± 386	1739 ± 402	1816 ± 606	1811 ± 489
Direct renin (pg mL^−1^)	11.7 ± 7.5	24.3 ± 22.7	10.3 ± 5.2	335.5 ± 544.4
Aldosterone (ng dL^−1^)	5.4 ± 4.7	11.8 ± 10.5*	4.2 ± 2.4	2.6 ± 0.7
Combination therapy	–	3 (2M/1F)	–	4 (2M/2F)

Values are means ± SD.

ABPM, ambulatory blood pressure monitoring; SBP, systolic blood pressure; DBP, diastolic blood pressure.

* Difference from pre-treatment within medication at *P *<* *0.05.

### Study design

After screening, patients who had been taking antihypertensive medications were weaned progressively from these drugs (“wash out period”). Thereafter, they were required to maintain a healthy lifestyle for 6 weeks according to the Eighth Report of Joint National Committee standard guidelines (James et al. 2014). Patients were required to monitor their BP daily using a Life Source BP monitor at home. They were also required to visit our Laboratory once per week for an office BP check during the run in period. If BP was ever above 160/100 mmHg, they were asked to resume their antihypertensive medications and were excluded from the study. Three subjects (2 men; 1 woman) were excluded based on this criterion. A 24-h ambulatory BP monitoring (ABPM, Ambulatory blood pressure monitoring; Oscar 2) was performed after the run in period. During the 24-h ABPM, BP was assessed every 30 min while awake and every hour during sleep.

Patients were randomly assigned to receive a medication with either the thiazide diuretic or the renin inhibitor for 6 months starting at a once daily low dose of 12.5 mg of HCTZ or 150 mg of ALSK. We increased the once daily dose to 25 mg (HCTZ) or 300 mg (ALSK) if BP was not well controlled (above 140/90 mmHg) within 2 weeks. If the high dose of monotherapy remained ineffective, a calcium channel blocker, amlodipine 5 mg once daily, was added the next month. Amlodipine was then increased to 10 mg once daily a month after the combination therapy was introduced if BP was still not well controlled. Medication was dispensed every 2 weeks and, therefore, patient compliance was assessed by the study nurse who counted the number of tablets remaining. Patients were tested before and after 6 months of antihypertensive medication treatment with these drugs. The 24-h ABPM was repeated prior to the post testing.

### Measurements

#### Muscle sympathetic nerve activity

Muscle sympathetic nerve activity signals were obtained using the microneurographic technique (Vallbo et al. 1979). Briefly, a recording electrode was placed in the peroneal nerve at the popliteal fossa and a reference electrode was placed subcutaneously 2–3 cm from the recording electrode. The nerve signals were amplified (gain 70,000–160,000), band-pass filtered (700–2000 Hz), full-wave rectified, and integrated with a resistance-capacitance circuit (time constant 0.1 sec). Criteria for adequate MSNA recording included: (1) pulse synchrony; (2) facilitation during the hypotensive phase of the Valsalva maneuver and suppression during the hypertensive overshoot after release; (3) increases in response to breath holding; and (4) insensitivity to a gentle skin touch or a loud shout (Vallbo et al. 1979).

#### Hemodynamics

Heart rate was determined from lead II of the electrocardiogram (ECG). Beat-by-beat arterial pressure (systolic BP, SBP; diastolic BP, DBP) was estimated noninvasively by using finger photoplethysmography (Nexfin, BMEYE, Amsterdam, the Netherlands). Brachial BP was obtained via electrosphygmomanometry (model 4240, SunTech Medical Instruments Inc., Raleigh, NC, USA).

### Protocol

For the 3 days prior to the studies, the subjects were placed on an isocaloric constant diet consisting of 100 mEq sodium, 100 mEq potassium, and 1000 mg calcium, while water intake was ad libitum. The experiment was performed ≥2 h after a light meal and ≥72 h after the last caffeinated or alcoholic beverage was consumed, and ≥24 h after strenuous physical activity. Experiments were conducted in a quiet, environmentally controlled laboratory with an ambient temperature of ˜25°C. The patient was placed in the supine position for intravenous catheter placement into the nondominant arm. After ≥30 min supine rest, a blood sample was withdrawn for baseline measurements of direct renin (radio-immunoassay) and aldosterone (chemiluminescent immunoassay). The plasma or serum was separated immediately by refrigerated centrifugation at −4°C and stored at −80°C until the assay.

At least 15 min after a satisfactory nerve recording site had been found, baseline (BL) data were recorded for 1 min. After that, the subject performed the cold pressor test (CPT). This was done by placing the subject's hand in an ice water slurry (2–4°C) for 2 min. During this time, the subject was asked to maintain a normal breathing pattern to avoid Valsalva maneuver-like activity (confirmed by nasal cannula). At the end of the 2 min, the subject's hand was removed from the ice water slurry, dried off, and placed between some warm towels (recovery, REC). We continued to collect REC data for 3 min. MSNA, heart rate (HR), and BP were recorded continuously. This protocol was used in our previous studies (Jarvis et al. 2011). After the REC period, the microneurography needles were removed.

Blood volume and red cell volume were measured using a modified carbon monoxide rebreathing method (Gore et al. 2006).

### Sample size calculations

Based on the results of Okada et al. (2013), we hypothesized that the ALSK group would demonstrate a blunted MSNA response during the CPT after treatment compared to the HCTZ group. We assumed that the mean difference between the groups was 11 bursts/min with standard deviation of 8 bursts/min. This indicated we would need 9 subjects per group to be able to reject the null hypothesis that the population means of the experimental and control groups were equal with a probability (power) of 0.80. The Type I error probability associated with this test of this null hypothesis was 0.05.

### Data analysis

Data were sampled at 625 Hz with a commercial data acquisition system (Acknowledge, Biopac Systems, Inc., Goleta, CA) and analyzed using LabView Software (National Instruments, Austin, TX) (Cui et al. 2001). Beat-by-beat HR was calculated from the R-R interval of the ECG. Beat-by-beat SBP and DBP were estimated from the arterial waveforms. MSNA bursts were identified from the integrated neurogram (Cui et al. 2001) and then were confirmed by trained personnel. Burst areas of the integrated neurogram and BP were measured simultaneously on a beat-to-beat basis. Burst frequency was defined as the number of bursts per min and burst incidence was used to normalize burst frequency per 100 heart beats. Total activity was defined as the burst area of the rectified and integrated neurogram. We assigned the largest burst amplitude during baseline a value of 100 (Sugiyama et al. 1996; Halliwill 2000). Therefore, all other bursts within a testing session were normalized against this value.

Muscle sympathetic nerve activity indices and hemodynamic variables were reported as a 1 min average for baseline (BL) and then analyzed as 30 sec epochs during the CPT and REC.

### Statistical analysis

Values are expressed as means ± SD. A two-way repeated measures analysis of variance [medication (HCTZ, ALSK) × phase (pre- vs. post treatment)] was used to compare subject characteristics, hemoglobin and hematocrit, blood and red cell volume, and the 24-h ABPM results.

A three-way repeated measures ANOVA [medication (HCTZ, ALSK) × phase (pre- vs. post treatment) × stage (BL, CPT, REC)] was used to examine hemodynamic and MSNA indices between medications, before and after treatment, and across stages. We also examined the difference from BL and minute 2 of the CPT (Δ) with a two-way repeated measures ANOVA to determine whether the magnitude of responses pre- and post treatment were comparable. Lastly, we used a Pearson correlation to determine whether a relationship between Δaldosterone (post-pre) and ΔMSNA existed, as well as between Δdirect renin and ΔMSNA. Tukey's post hoc analysis was used when significance was found. All statistical analyses were performed using SigmaPlot 12.5 (Systat Software Inc., San Jose, CA). A *P*-value of <0.05 was considered statistically significant.

## Results

### Subject characteristics

Subjects in both the HCTZ and ALSK groups were well matched for physical characteristics (Table 1). Both medications comparably decreased SBP during the awake and sleep periods (*P *<* *0.05). The awake DBP was also lowered with both treatments (*P *<* *0.05); however, only the HCTZ group demonstrated a decrease in the sleep DBP after treatment (*P *<* *0.05). There were no differences between groups in hematocrit, hemoglobin, total blood volume, or red cell volume. There was, however, a main effect of hematocrit (*P *<* *0.05), which was lower after 6 months of antihypertensive treatment. Direct renin tended to be higher in the ALSK group (*P *=* *0.097) and after 6 months of treatment (*P *=* *0.081). Aldosterone was higher in the HCTZ group after treatment (*P < *0.001). The numbers of subjects that received combination therapy (HCTZ or ALSK and amlodipine) are also illustrated in Table 1.

### Hemodynamic responses to the CPT

**Figure 1:**
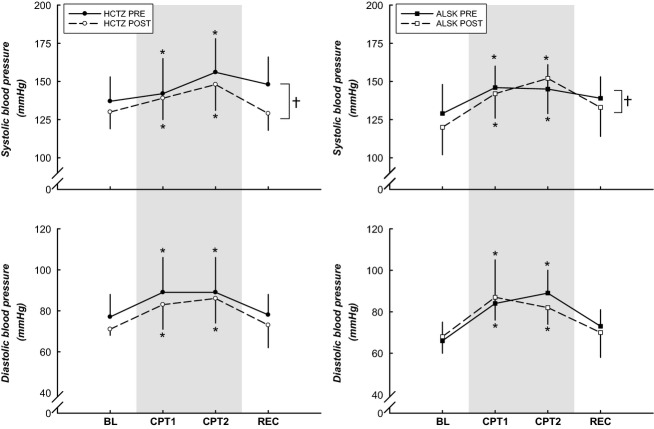
BP responses to the CPT. Both groups demonstrated increases in SBP and DBP in response to the CPT. SBP was lower after the 6 month medication intervention. *Difference from BL, *P *<* *0.05. †Difference from pretreatment, *P *<* *0.05. Data are presented as mean ± SD.

**Figure 2:**
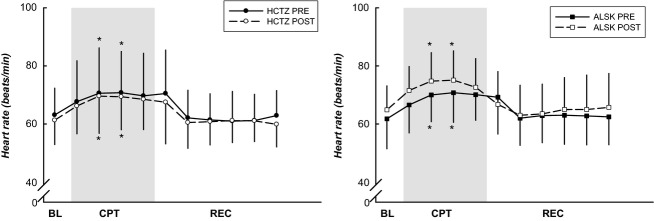
HR responses to the CPT. HR was elevated during minute 1–1.5 of the CPT when compared to BL. Subjects on ALSK showed a trend toward a higher HR during the post-treatment compared to pretreatment (*P *=* *0.064). *Difference from BL, *P *<* *0.05. Data are presented as mean ± SD.

As depicted in Figure 1, both groups demonstrated increases in SBP and DBP in response to the CPT. SBP was lower (*P *<* *0.05) after the 6 month medication intervention. HR (Fig. 2) was elevated during minute 1–1.5 of the CPT when compared to BL in both groups (*P *<* *0.05). Subjects on ALSK showed a trend toward a higher HR (*P *=* *0.064). There was also a trend toward a higher HR after 6 months of treatment in the ALSK group (medication × stage interaction, *P *=* *0.071).

Tables2 and 3 illustrate the magnitude of the hemodynamic responses from BL to CPT (peak CPT, minute 2 of the CPT) and BL to end REC before and after treatment, between the two antihypertensive medication groups. Before treatment, SBP was lower during the end REC phase (−7 ± 13 and −5 ± 12 mmHg for HCTZ and ALSK). However, after treatment this response was abolished (1 ± 9 and 5 ± 9 mmHg for HCTZ and ALSK, *P *<* *0.05 from pretreatment). All other hemodynamic responses were similar pre- and post treatment.

**Table 2:** Changes from BL to peak CPT and end REC for the HCTZ group

	PRE		POST	
	Peak CPT	End REC	Peak CPT	End REC
SBP (mmHg)	26 ± 10	−7 ± 13	17 ± 21	1 ± 9*
DBP (mmHg)	15 ± 8	−3 ± 4	9 ± 13	3 ± 7
HR (beats/min)	7 ± 8	−2 ± 3	7 ± 8	−1 ± 2
MSNA-BF (bursts/min)	13 ± 8	−3 ± 20	11 ± 11	2 ± 6
MSNA-BI (bursts/100 heart beats)	14 ± 9	−2 ± 34	9 ± 16	3 ± 9
MSNA Amplitude (a.u.)	1912 ± 1406	68 ± 1062	1402 ± 1077	−60 ± 324
MSNA-Total activity (a.u./min)	458 ± 314	24 ± 260	376 ± 308	10 ± 126

Values are mean ± SD.

Peak CPT was defined as minute 2 of the CPT.

* Difference from pretreatment within medication at *P *<* *0.05.

**Table 3:** Changes from BL to peak CPT and end REC for the ALSK group

	PRE		POST	
	Peak CPT	End REC	Peak CPT	End REC
SBP (mmHg)	21 ± 20	−5 ± 12	29 ± 15	5 ± 9*
DBP (mmHg)	13 ± 11	0 ± 8	12 ± 6	0 ± 5
HR (beats/min)	8 ± 7	1 ± 5	8 ± 5	1 ± 7
MSNA-BF (bursts/min)	11 ± 17	−5 ± 9	6 ± 13	−7 ± 17
MSNA-BI (bursts/100 heart beats)	10 ± 21	−6 ± 14	1 ± 13	−10 ± 20
MSNA Amplitude (a.u.)	1684 ± 1812	−65 ± 504	947 ± 1656	−453 ± 1120
MSNA-Total activity (a.u./min)	422 ± 442	−12 ± 82	207 ± 401	−103 ± 300

Values are mean ± SD.

Peak CPT was defined as minute 2 of the CPT.

* Difference from pretreatment within medication at *P *<* *0.05.

### Sympathetic neural activity responses to the CPT

**Figure 3:**
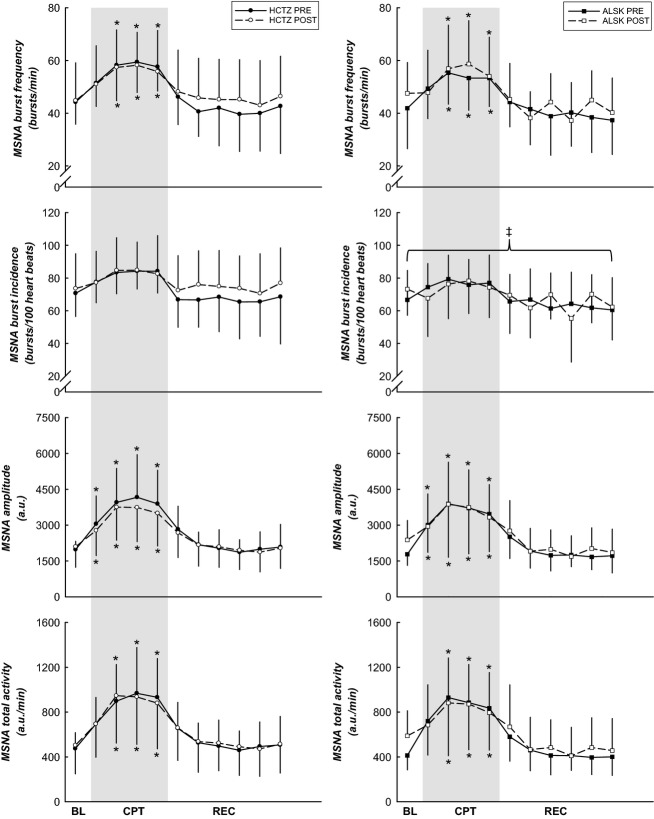
MSNA responses to the CPT. MSNA burst frequency, amplitude, and total activity were higher during the CPT when compared to BL. MSNA burst incidence was higher in the HCTZ treatment group. *Difference from BL, *P *<* *0.05. &ddagger;Difference between treatment groups, *P *<* *0.05. Data are presented as mean ± SD.

Muscle sympathetic nerve activity burst frequency, amplitude, and total activity (Fig. 3) were higher during the CPT when compared to BL (all *P *<* *0.05, both medication groups). There was a trend for the main effect of MSNA burst frequency to be higher in the HCTZ group (*P *=* *0.068). The main effect of MSNA burst incidence (Fig. 3) was higher in the HCTZ treatment group than in the ALSK group (*P *=* *0.001). MSNA amplitude and total activity were comparable between the two medications (*P *>* *0.05).

Tables2 and 3 illustrate the magnitude of the MSNA responses from BL to CPT (peak CPT, during minute 2) and from BL to end REC, before and after treatment between the two antihypertensive medication groups. There were no differences in the magnitude of the responses before or after treatment in either the HCTZ or ALSK groups (all *P *>* *0.05).

### Sympathetic neural activity and hormonal interactions

There was no relationship between changes in aldosterone and direct renin and changes in MSNA burst incidence or total activity (*P *>* *0.05).

## Discussion

The major findings from this study are: (1) HCTZ and ALSK were equally effective in lowering awake SBP and DBP from the 24-h ABPM after 6 months of medication treatment; (2) SBP, DBP, and HR responses were similar between groups in response to the CPT; (3) MSNA burst frequency (main effect) tended to be higher and MSNA burst incidence (main effect) was higher in the HCTZ group; and (4) all MSNA indices demonstrated similar changes in response to the CPT between the two groups. These results demonstrate that the magnitude of the responses to a CPT remain similar before and after 6 months of medication, despite adequate control of blood pressure during this period. That is, subjects maintained hyperreactivity to the CPT even after treatment with HCTZ and ALSK. Contrary to our hypothesis, treatment with a renin inhibitor did not depress sympathetic neural control during a CPT when compared to those taking a thiazide diuretic. The similarity in the magnitude of the responses (peak CPT and end REC) between pre- and post-treatment indicate that central integration and the efferent pathways were unaffected by these medications. These findings may lend insight into the unfavorable cardiovascular outcomes in hypertensives, despite having well controlled BP (Ivanovic et al. 2004).

### Chronic effects of thiazide diuretics on sympathetic neural control during the CPT

Okada et al. (2013) reported that treatment with a thiazide diuretic enhanced sympathetic activity during upright tilt. The current study showed a trend toward higher burst frequency and a significantly higher burst incidence in the HCTZ group. As this difference is a main effect, we interpret this finding as chance subject selection. That is, by random selection the group receiving HCTZ had higher overall MSNA.

We found that HCTZ increased the contributions of RAAS, consistent with others (Okada et al. 2013). Aldosterone was elevated in the HCTZ group after 6 months of treatment suggesting a compensatory increase in products of the RAAS to counterbalance the diuretic effect. Aldosterone by itself does not elicit a sympathoexcitatory effect; however, the increase in aldosterone was likely stimulated by an increase in angiotensin II (not directly measured). If this is true, it is possible that the increase in angiotensin II after HCTZ treatment might cause a sympathoexcitatory response through central mechanisms (Gao et al. 2005). However, the MSNA response was not increased during the CPT after treatment, probably because 2 min was too short to elicit the activation of the RAAS. It is also possible that sympathetic activity was increased in another circulation such as the renal bed or splanchnic region.

Our findings are consistent with the limited number of studies that have previously examined the influence of thiazide diuretic treatment and responses to the CPT (Eliasson et al. 1986, 1987). While these studies did not examine MSNA responses, one reported that BP responsiveness was the same before and after 6 months of hydrochlorothiazide treatment (Eliasson et al. 1987). Their subject population was dissimilar to ours; their subjects were younger (˜40-years old) and required a higher dose of HCTZ (50 mg) to control BP (Eliasson et al. 1987). However, our findings combined with those of Eliasson et al. (1987) underscore that responsiveness to stressors should be an important consideration when treating those with hypertension.

### Chronic effects of renin inhibition on sympathetic neural control during the CPT

Baseline MSNA and responses to the CPT were not depressed after treatment in this group as we hypothesized. While the CPT is a relatively short manipulation of BP we surmised that the chronic effects of the renin inhibition prior to the CPT would have led to an overall blunting of the sympathetic response. That is, based on previous studies, through the reduction in renin and, consequently, angiotensin II we expected to see an overall reduction in sympathetic outflow (Murakami et al. 1997; Gao et al. 2005). Our findings are among the first to demonstrate that renin inhibition does not influence central integration and the efferent pathway of neural control of BP during acute hypertensive manipulations, such as the CPT.

This differs from what we (Okada et al. 2013) observed during upright tilt as we reported that renin inhibition (ALSK) was more effective in lowering the sympathetic response. This is likely explained by the length of time of the stimulus. Upright tilt (>15 min) activates the RAAS such that the therapeutic effect of ALSK is more obvious. A very acute stimulus (2 min), such as the CPT, is too short to elicit renal-adrenal hormone release; therefore, the impact of ALSK treatment cannot be seen.

### Clinical implications

Cardiovascular responses to the CPT have been used as a predictor of the future development of hypertension (Murakami et al. 1980; Wood et al. 1984). Exaggerated responses have also been observed in patients with coronary artery disease when compared to those experiencing chest pain but without coronary artery disease (Sevre and Rostrup 1999). More recently, these BP responses have been shown to predict carotid-femoral pulse wave velocity progression, an index of arterial stiffness, in men over a 5 year span (Bellinazzi et al. 2014). Bellinazzi et al. (2014) hypothesized that the same relationship was not found in women because of blunted vascular transduction in women. We can support this hypothesis with data from our own Laboratory (unpublished) and in previous reports from others examining various circulations (Kneale et al. 2000; Jarvis et al. 2010). However, from a clinical perspective, our findings and those of Bellinazzi et al. (2014) suggest that, despite adequate control of BP, the pathophysiologic progression of arterial stiffness remains present in this patient population. This may be one explanation for the unfavorable cardiovascular outcomes in those with hypertension. Therefore, treatment strategies should not only focus on the reduction of BP but should also strive to decrease the sympathoexcitatory drive during physiological stimuli.

### Limitations

One limitation is that a subset of subjects received combination therapy with amlodipine during the study. Three subjects in the HCTZ group and four from ALSK were not adequately controlled with monotherapy. We acknowledge this is a limitation in interpreting our results as previous studies have indicated that treatment with calcium channel blockers may invoke a baroreflex mediated increase in sympathetic activity, as previously assessed by norepinephrine spillover (Lindqvist et al. 1994). However, we also outlined this as likely to be one of the mechanisms for trend in increased MSNA observed in the HCTZ group; therefore, we do not believe that treatment with combination therapy altered the findings or interpretation as neither group demonstrated different responses from pre- to post- treatment.

Second, the CPT is a relatively short manipulation of BP and therefore medications like a renin inhibitor may have no impact on these responses because of the relationship between the RAAS and more long-term BP control. However, the results from this study still provide insight into the unfavorable cardiovascular outcomes in those with hypertension, even with adequate control of BP.

In conclusion, thiazide diuretics and renin inhibitors are both successful ways of lowering BP in elderly mild hypertensive patients. However, neither treatment with HCTZ or ALSK altered sympathetic outflow. The magnitude of the responses was similar between the groups and after treatment when exposed to a CPT. These findings indicate that sympathetic neural outflow and responses remain similar even during successful treatment of hypertension. The persistent sympathetic hyperreactivity to stressors may be one explanation for the unfavorable cardiovascular outcomes in hypertensives despite adequate control of BP.
